# Spatial analysis to support geographic targeting of genotypes to environments

**DOI:** 10.3389/fphys.2013.00040

**Published:** 2013-03-18

**Authors:** Glenn Hyman, Dave Hodson, Peter Jones

**Affiliations:** ^1^Centro Internacional de Agricultura Tropical (CIAT)Cali, Colombia; ^2^International Maize and Wheat Improvement Center (CIMMYT)Addis Ababa, Ethiopia; ^3^Waen AssociatesGwynedd, UK

**Keywords:** spatial analysis, genotype-by-environment interaction, geographic targeting

## Abstract

Crop improvement efforts have benefited greatly from advances in available data, computing technology, and methods for targeting genotypes to environments. These advances support the analysis of genotype by environment interactions (GEI) to understand how well a genotype adapts to environmental conditions. This paper reviews the use of spatial analysis to support crop improvement research aimed at matching genotypes to their most appropriate environmental niches. Better data sets are now available on soils, weather and climate, elevation, vegetation, crop distribution, and local conditions where genotypes are tested in experimental trial sites. The improved data are now combined with spatial analysis methods to compare environmental conditions across sites, create agro-ecological region maps, and assess environment change. Climate, elevation, and vegetation data sets are now widely available, supporting analyses that were much more difficult even 5 or 10 years ago. While detailed soil data for many parts of the world remains difficult to acquire for crop improvement studies, new advances in digital soil mapping are likely to improve our capacity. Site analysis and matching and regional targeting methods have advanced in parallel to data and technology improvements. All these developments have increased our capacity to link genotype to phenotype and point to a vast potential to improve crop adaptation efforts.

## Introduction

From the 1960s to the 1980s, the Centers of the Consultative Group on International Agricultural Research (CGIAR) pursued research on genotype by environment interactions (GEI) research with relatively unfettered budgets. Their mandate was to produce new crop varieties, train people to use them, and get the seeds to the world's farmers. To this end, the Centers produced great networks of testing sites all over the world (see, e.g., Peterson and Pfeiffer, 1989; CIAT, 2001; Magorokosho et al., 2007). Collaborators came from national government breeding programmes and universities, wherever there was an interest. Many of the results are archived, and can be very useful in studies of GEI and in suggesting where a new genotype might fit. The collapse of the Berlin Wall saw a new era in funding for international agricultural research and development (Pardey et al., 2006), with developed nations reducing their contributions. The world's food supply problems were no longer of geopolitical importance. Accordingly, the international cultivar testing programmes have declined, hindering our capacity to supply farmers with improved varieties adapted to their environments.

While crop improvement programmes are faced with reduced funding for agricultural research, the use of models, maps and computer tools can help boost efficiency in their development and testing of cultivars for dissemination to farmers. Cultivar testing and dissemination programmes need spatial analysis to help target genotypes to environments. By supporting GEI assessments, maps and models can predict how well cultivars will respond to particular environments. Ultimately, spatial analysis can help in the dissemination of varieties to the farmers that need them.

How can spatial analysis be used to help breeders decide where to test and disseminate varieties? Systematic sampling of sites can help ensure coverage of a diverse range of environments where farmers may take up the cultivar. A site testing design focusing on one or several environments but leaving out many others could miss areas where the cultivar might produce high yields. A breeding programme with a fixed budget for testing may also want to avoid duplication of sites in similar environments.

Several advances over the last few decades have improved the capacity to apply spatial analysis to phenotyping and GEI analysis. Vital to all these advances has been the development of computer hardware and software that has allowed many types of analysis that were impossible to carry out before. Advances in weather and soil monitoring instruments have improved data collection, and a key resource for spatial analysis in agriculture has been the availability of climate data in digital formats. Global soil mapping efforts have been slow to develop—with little attention prior to 1950. New soil mapping methods have improved on the standard Food and Agriculture Organization (FAO) global soil base map at 1:5 million scale (FAO, 1996, 2008b). Agricultural censuses and surveys have also added to the set of data resources available. Related to these baseline datasets are derived data such as climate maps, crop distribution surfaces, and socioeconomic information. These advances have led to agro-ecological zoning maps (Bunting, 1987), weather generators (Hartkamp et al., 2003), and sophisticated statistical analysis of GEI (e.g., Crossa et al., 2004; Setimela et al., 2005). More recently, combinations of crop simulation models and geographic information systems (GIS) have improved our understanding of spatial and temporal aspects of GEI (e.g., Loffler et al., 2005).

This paper reviews and discusses the development of spatial analysis for crop improvement and how it can be used to increase the efficiency of testing and deployment of genotypes. First, advances in the development of spatial data for agricultural applications are discussed, followed by how spatial analysis, and GIS can be used to support geographic targeting of genotypes to environments. The discussion includes the development of agro-ecological maps and environmental change considerations in crop improvement efforts. The paper concludes with a discussion of trends in the use of spatial databases and GIS in crop improvement programmes. Throughout the paper, references are made to data, tools, and resources for applying spatial analysis to crop improvement.

## Advances in spatial data

Several types of spatial analysis for crop improvement as conducted today would have been difficult to carry out even 5 or 10 years ago. Perhaps the greatest advances have come in mapping climate, although information on soils and other environmental parameters is now much more widely available than in the past. Advances in data availability have substantially increased the potential for spatial analysis to support the planning and assessment of phenotyping and variety trials. Assessments should ensure that a sufficient range of environments is tested, so as to adequately study GEI. Improvements in data have more than kept pace with advances in the methodology of spatial analysis for phenotyping. This section surveys data development for spatial analysis, and serves as a guide to spatial data acquisition for the agricultural scientist using GIS for phenotyping. Table 1 lists some key spatial data sets that are publicly available and can be used in crop improvement efforts.

**Table 1:** **Key spatial data sets that are publicly available**.

**Source**^a^	**Application**	**Resolution**	**URL**
FAO SOIL	Soil analysis	1:5m	http://www.fao.org/nr/land/soils/digital-soil-map-of-the-world/en/
ISRIC	Soil analysis	n/a	http://www.isric.org/UK/About+Soils/Soil+data/
HWSD	Soil analysis	n/a	http://www.iiasa.ac.at/Research/LUC/luc07/External-World-soil-database/HTML/
WISE	Soil profile analysis	n/a	http://www.isric.org/UK/About+ISRIC/Projects/Track+Record/WISE.htm
CRU	Climate	0.5°	http://www.cgiar-csi.org/data/climate
IWMI World Water Atlas	Climate; hydrology	Various	http://www.iwmi.cgiar.org/WAtlas/
NOAA	GSOD^b^	Point data	http://www.ncdc.noaa.gov/oa/gsod.html
Worldclim	Climate	1 km	http://www.worldclim.org
NASA POWER	Climate	1°	http://power.larc.nasa.gov/
TRMM	Tropical rainfall	0.25°	http://trmm.gsfc.nasa.gov/
SRTM	Elevation	90 m	http://srtm.csi.cgiar.org/
AgroMaps	Crop distribution	n/a	http://www.fao.org/landandwater/agll/agromaps/interactive/page.jspx
Globcover	Land cover	300 m	http://ionia1.esrin.esa.int/
Biogeomancer	Gazetteer	n/a	http://www.biogeomancer.org/

a ISRIC, International Soils Reference and Information Center; HWSD, Harmonized World Soil Database; WISE, World Inventory of Soil Emission Potentials; CRU, Climate Research Unit of the University of East Anglia; IWMI, International Water Management Institute; NOAA, National Oceanic and Atmospheric Administration; NASA POWER, National Aeronautics and Space Administration Prediction of World Energy Resource; TRMM, Tropical Rainfall Measuring Mission; SRTM, Shuttle Radar Topography Mission.

b GSOD, Global Surface Summary of the Day.

### Soils

Data on soil properties are a key category of information for agro-ecological assessments. However, advances in the development of soil datasets are hindered by the difficulty of mapping the entire world. The main problem is that soils can be highly variable even across short distances. Moreover, not all countries use the same soil classification systems. The concept of the likelihood or probability of finding a given soil property has been used to reflect data uncertainty at a particular point when using maps like the FAO 1:5 million soil map of the World (FAO, 1996, 2008b). This map remains the most widely used soil map for continental and global applications. Sanchez et al. (2003) derived soil constraint data in the context of the Fertility Capability Classification, based on this FAO map. The International Soils Reference and Information Center (ISRIC) also used the FAO soil map, adding soil profile information to develop the World Inventory of Soil Emission Potentials (WISE) database of derived soil parameters (e.g., pH, drainage, organic carbon content) for the world at 5 arc minute resolution (Batjes et al., 2007; Batjes, 2009). However, an initiative is underway to develop the Harmonized World Soil Database (FAO et al., 2008). The project aims to merge different soil maps and produce a new global map at a 1:1 million scale. To date, the effort includes FAO's regional Soil Terrain Database studies (SOTER; FAO, 1995), the European Soil Database and the Soil Map of China. The main gaps that need to be filled include Central and West Africa, the Middle East and South Asia. This data set should be updated in the coming years as new data become available. Finally, CIAT (2009) has initiated the production of a digital soil map of Africa. This new effort aims to create high-resolution maps of better quality, based on innovations in the remote sensing of soil properties and the management of geographic information.

### Climate

Phenotyping programmes and GEI assessments can benefit from broad-scale climate analysis to assess to what extent sites represent target environments. An important recent advance in climatic analysis is the availability of ready-to-use climate data available over the Internet or in software applications. Acquiring climate data depended in the past on contacts between researchers who developed climate datasets. The overall quantity of weather station data has dropped compared to past decades (Ramirez-Villegas and Challinor, 2012). More recently, software tools such as CIAT's FloraMap®, Homologue and MarkSim® provide climate data associated with specialized applications (Jones and Thornton, 1993, 2000; Jones et al., 2002, 2007a). Other climate tools include some of FAO's standard data CD-ROMs and applications, such as their Local Climate Estimator (LocClim), and datasets on CD-ROM from the International Water Management Institute (IWMI) (FAO, 2005; IWMI, 2008). While some of these tools lack the capability to extract global or regional climate surfaces, they were the first to provide broad-scale climate data for agricultural science applications.

Two relatively new sources of data on the Internet have broadened the capacity to incorporate climate information in spatial analysis applications for agriculture. The University of East Anglia's Climate Research Unit data include key variables needed for climate analysis, such as rainfall, temperature, relative humidity, wind direction and speed, among others (New et al., 2002; CGIAR-Consortium for Spatial Information (CGIAR-CSI), 2006). Another important data source is Worldclim (Hijmans et al., 2005), which includes precipitation and temperature data available at spatial resolutions of 1 km and coarser. Worldclim has also derived some data sets from precipitation and temperature variables, including information on seasonality, temperature ranges, and climate conditions in the wettest, driest, coldest, and warmest months and quarters (Busby, 1991). Both of these datasets draw on spatial interpolation methods to estimate climate parameters between weather stations.

Climate datasets derived from remote sensing hold some promise for use in agro-ecological assessment. The Moderate Resolution Imaging Spectroradiometer (MODIS) satellite platform includes surface temperature data (NASA, 2008a). Rainfall estimates (RFE) from satellite-based datasets are now widely available. The Tropical Rainfall Measuring Mission (TRMM) provides RFE for 3-h time periods for much of the world (NASA, 2008b). The Climate Prediction Center MORPHing technique (CMORPH) dataset (Joyce et al., 2004) provides 3-hourly RFE globally at a spatial resolution of 0.25°. The RFE dataset from the National Oceanic and Atmospheric Administration (NOAA)/Climate Prediction Center (CPC) provides daily data at a spatial resolution of 8 km (Herman et al., 1997; Xie et al., 2002). These data have yet to be verified and validated to the point that they are widely used for agro-ecological assessments, although it should be noted that the RFE data form the basis of several famine early-warning products^1^ and FAO routinely uses the CMORPH data in monitoring Desert Locusts^2^. Combining ground weather data with remotely sensed information will be a key area of research in the future.

### Elevation

Elevation is another important data set for spatial analysis in agriculture and can be used to help establish the ecological niche of a genotype. It can be used as an auxiliary variable in assessing climate or in analysing the role of topography in agriculture. Until recently, global digital elevation models were derived from 1:1 million mapping efforts, such as the Digital Chart of the World (ESRI, 1992). The now widely available Shuttle Radar Topography Mission data set has 90 m spatial resolution, the best available from coverage of the whole land surface (Jarvis et al., 2004; CGIAR-CSI, 2008).

### Vegetation and crop geography

Vegetation and crop geography assessments can be made from remote sensing data, censuses, and surveys, and from combinations of these. Remote sensing platforms provide vegetation data as an additional dataset for agro-ecological characterization, even though it has rarely been used in classification to date. The Advanced Very High Resolution Radiometer (AVHRR) and MODIS satellite platforms provide 1-km resolution data sets going back to 1980. Satellite data at finer resolutions can also produce vegetation data. The most common variables are the normalized difference vegetation index (NDVI) and the enhanced vegetation index (EVI).

Land cover maps derived from remotely sensed data can be used to match potential crop environments with areas classified as croplands. Several broad-area assessments have been conducted. These include GeoCover, GLC2000 and Globcover (Bartholome and Belward, 2005; Bicheron et al., 2006; Arino et al., 2008). Wood et al. (2000) developed a map of cropland intensity for the year 2000, showing the percentage of a grid cell with cropland. The Globcover dataset, a 2005 snapshot of land cover at 300 m resolution, is the most recent global land cover product. While global land cover datasets all have their shortcomings with respect to accuracy and discrimination of land cover types, their increasing availability will lead to their increased use for agricultural applications.

Research and development efforts have produced several important datasets on the geography of key staple crops, including cassava (Carter, 1987; Carter et al., 1992), sweet potatoes [International Potato Center (CIP), 2006a,b, Hijmans, 2001; Hijmans et al., 2001], beans (Wortman et al., 1998), maize (Hodson et al., 1999), rice (Huke and Huke, 1997; Robison et al., 1984), and wheat (Lantican et al., 2005; Hodson and White, 2007), among others. Unfortunately, these crop-specific mapping efforts often lack comparability between crops. They may have widely different temporal and spatial frameworks, as well as using different methods to produce the datasets. While this lack of standardization does not necessarily affect genotype targeting efforts, standardized initiatives would go a long way toward improving the quality of data for analysis.

Mapping programmes that include multiple crops could take advantage of a common set of standards in data development. Drawing on the support of United Nations (UN) member countries, FAO's AgroMaps programme aims to map sub-national agricultural production data from agricultural censuses and surveys (FAO, 2008a). FAO plans to link the effort with national level statistics from FAOSTAT—something that could improve the quality of both datasets. Other efforts map sub-national agricultural production at global, regional, and local levels but AgroMaps is the only one that makes its data freely available on the Internet. An inspection of the number of crops and the resolution of administrative districts points out some substantial limitations of AgroMaps—problems that will be difficult to overcome without greater international efforts to promote agricultural census-taking.

A recent trend in crop mapping is the combination of survey and census data with remote sensing information. Crop production data can be converted to grid cell maps to more precisely characterize the spatial distribution of the crop (e.g., Leemans and Van Den Born, 1994; Ramankutty and Foley, 1998; Leff et al., 2004; Ramankutty, 2004; You and Wood, 2006; You et al., 2009). The conversion allocates production to small grid cells where the likelihood of the presence of the crop is greatest, eliminating forest, urban, pasture and other types of land cover where we would not find the crop. While the conversion of production data to a grid cell framework raises concerns with ecological fallacy and the modifiable areal unit problem (MAUP), the coarseness of most production datasets requires grid maps (Openshaw, 1984; Freedman, 1999). Improving these grid maps requires, first and foremost, better input data. Researchers need greater spatial and temporal resolution of crops statistics and remotely sensed data. Even so, there is great scope for improving allocation algorithms used in making grid cell maps.

### Trial sites data

Efforts to target genotypes to environments may also take advantage of locating genetic resources data in terms of their respective development and testing sites or the location of pedigree accessions, including wild relatives of food crops (Jarvis et al., 2005). Many genebanks lack well-documented information on the spatial location of the materials they manage (Hijmans et al., 2000). When genetic resources data do have coordinate information, it is often incorrect, requiring an effort to georeference the data (Hijmans et al., 1999; Biogeomancer, 2007). Several efforts are now underway to address these issues and provide improved access to georeferenced genebank data. The Focused Identification of Germplasm Strategy (FIGS) system for Bread Wheat accessions is one example^3^.

Maps of variety trial sites are essential for linking phenotyping to spatial analysis. International yield trials networks, such as the bean, *Musa*, maize and wheat initiatives (Peterson and Pfeiffer, 1989; Jones and Tezenas du Montcel, 1994; CIAT, 2001), have tended to develop reasonably good maps of their trial networks. Usually, the locations of trial sites are held outside of the public domain. In many cases, information on these trial sites is outdated or poorly documented. Another problem is that the location information is often imprecise, leading to the generation of errors in spatial analysis.

The greatest deficiency with respect to trial site data is the lack of weather and soil information. In some cases, these data simply were not collected. In other cases, they remain unpublished, either in journal publications or in gray literature. One solution to acquire these data is to find them through international climate and soil databases, either in GIS formats or by locating the nearest point location. For example, the WISE database may include some soil profiles taken from experiment stations where trials are conducted. Station climate data from NOAA's Global Surface Summary of the Day (GSOD)^4^ can be used to match a site to the nearest site to a weather station. Weather information for any site could also be acquired from the National Aeronautics and Space Administration (NASA) Prediction of World Energy Resource (POWER) dataset, which provides daily rainfall and temperature data for the last 12 years (NASA, 2009). However, using secondary data from GIS databases—data that was not actually derived at the trial site—may increase errors substantially. Whenever these secondary data are used, the researcher should mention its' reliability and should include any available error estimates.

A whole series of other socioeconomic data sets could be used to target genotypes to environments. These might include human population data sets (Center for International Earth Science Information Network (CIESIN) et al., 2004), accessibility and transportation infrastructure data (Nelson, 2008), and human welfare data (e.g., CIAT, 2006). However, these would be useful more for logistics planning of germplasm deployment, rather than for testing. Sites with high rural populations and accessibility and with substantial poverty may be attractive relative to isolated sites outside of areas that would be likely targets for variety dissemination. These types of data could be used when a breeding programme is near the end of the variety development cycle, to search for sites that can be used to support germplasm deployment. Building on efforts since the 1980s to collect this type of information, dedicated programmes aimed at global mapping have improved the availability of these socio-economic and agricultural production data.

## Siting and regional targeting of genotypes

Targeting genotypes to environments has developed substantially since the middle of the last century. Early breeding efforts led scientists to use their knowledge of a crop to speculate on how well their varieties might perform in new locations, and they could experiment in a range of sites to test GEI. Eventually, international trial networks were set up to provide scientifically rigorous testing regimes (e.g., Peterson and Pfeiffer, 1989; CIAT, 2001).

Most early breeding efforts sought to develop cultivars with wide adaptation (e.g., Braun et al., 1996). Later efforts aimed to target niche environments with unique abiotic or biotic stresses (e.g., Wilhelmi et al., 2002; Annicchiarico et al., 2005). The latter approach led to greater demand for mapping the agro-ecology of a crop, supporting the breeders' targeting of a genotype to specific conditions.

An understanding of the target environment and the extent of GEI are essential elements of all breeding programmes. GEI take several forms but of major concern are the crossover interactions, where the GEI result in a change in the rank of the genotypes between environments and hence influence the nature, magnitude, and predictability of the selection response achieved by any breeding programme (e.g., Cooper, 1999).

Using multienvironmental trials, breeders draw on statistical techniques developed to measure GEI (Finlay and Wilkinson, 1963). The statistical tools developed have centered on the use of 88 linear–bilinear models and mixed models (Crossa et al., 2004), and have permitted a better understanding of crossover GEI. These tools permit the identification of clusters of sites or genotypes that show little or no crossover GEI. As a result, a smaller number of globally representative key locations can be identified that assist breeders in the selection of widely adapted germplasm. Ultimately, these statistical methods and GIS can be used to recommend cultivars for specific locations (Annicchiarico et al., 2005, 2006).

For wheat, analyses of several major international trial nurseries of CIMMYT (Centro Internacional de Mejoramiento de Maiz y Trigo; International Maize and Wheat Improvement Center) have been undertaken using these statistical approaches (e.g., Trethowan et al., 2001, 2002, 2003; Lillemo et al., 2005). Analysis of sites and variety performance builds on an extensive literature related to multienvironment trial networks (DeLacy et al., 1996; van Eeuwijk et al., 2001).

Plant breeders can use soil and climate information of the trial sites to classify these point locations into more or less homogenous environment types (DeLacy et al., 1994; Mgonja et al., 2002; Setimela et al., 2003, 2005; Maideni, 2006; Roozeboom et al., 2008). Grouping trial sites can be useful in designing field testing plans for plant breeding programmes, but may not tell us ultimately where genotypes can perform well because the sites only represent a limited number of point locations. Therefore, linking individual trials sites to larger regions for which they are representative opens up numerous possibilities for phenotyping work and, ultimately, for introducing varieties into environments where they are expected to perform well (DeLacy et al., 1994; Gauch and Zobel, 1997). The following sections discuss environment-matching methods and crop-specific agro-ecological mapping, and their use for targeting genotypes to environments.

### Site analysis and matching

Environmental data on the sites of variety trials or potential future trials can give us key information for targeting genotypes to environments. Any number of sites can be compared to each other to determine their similarity in terms of climate and soils. Researchers may use a number of different methods to make these comparisons. A few examples are given here to illustrate some of the issues in comparing sites.

**Figure 1:**
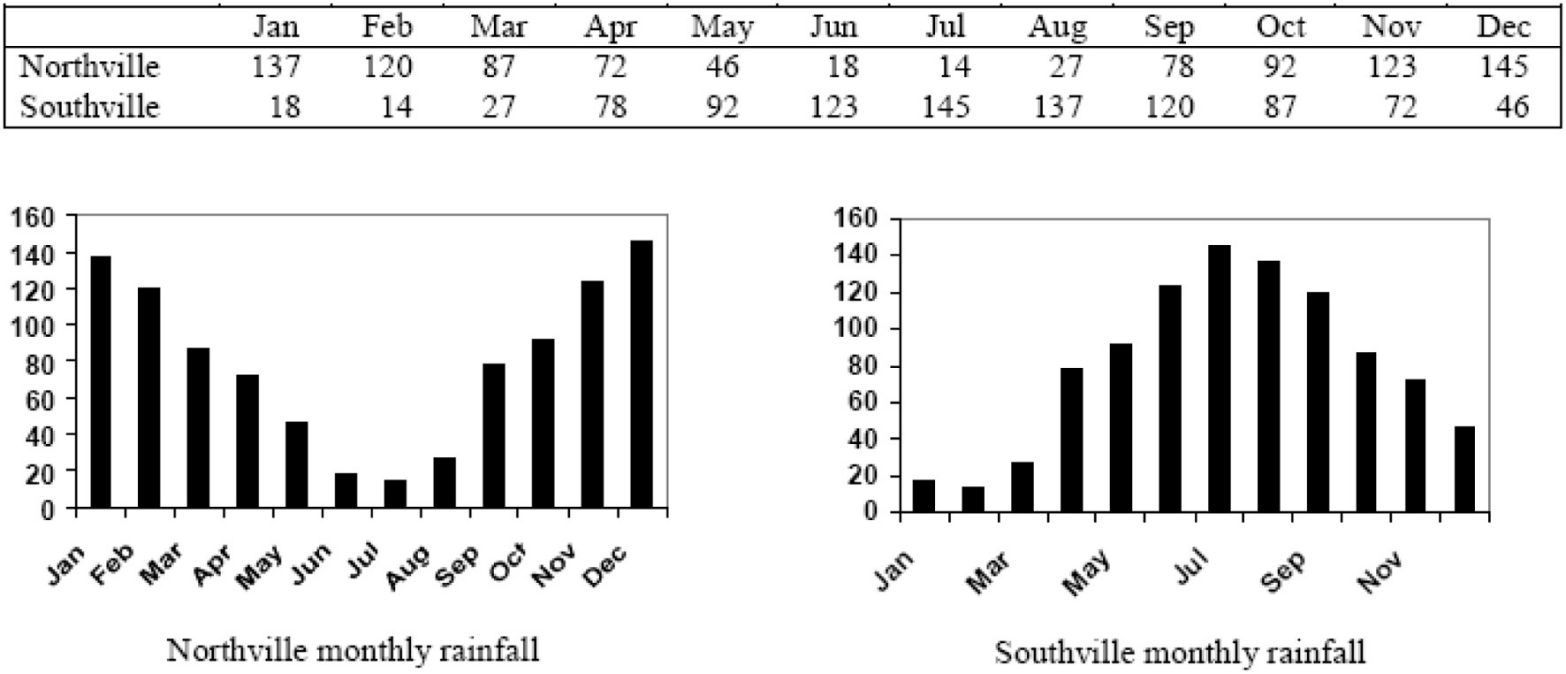
**Two hypothetical pluviographs exhibiting identical rainfall patterns (Source: http://gisweb.ciat.cgiar.org/marksim/)**.

Measuring site similarity requires methods to be able to compare climate data at different locations. Since climates vary with latitude and season, similar levels of rainfall or temperature can occur at different times of the year. One way to account for these differences is to express climate data in terms of their relationship to climate extremes, removing reference to the date of the data. For example, the BIOCLIM method uses data on rainfall and temperatures in the wettest, driest, warmest, and coldest months (Busby, 1991). A more common method is to transform the data to “standard” time scale. Figure 1 illustrates rainfall of a hypothetical climate in the northern hemisphere and an identifical one in the southern hemisphere. In order to standardize these climate patterns, Jones and Thornton (1993) describe a 12-point Fourier transform to rotate the data to a standard season. Other methods are variations on the same process of standardizing the seasons so that climates at different latitudes can be compared.

**Figure 2:**
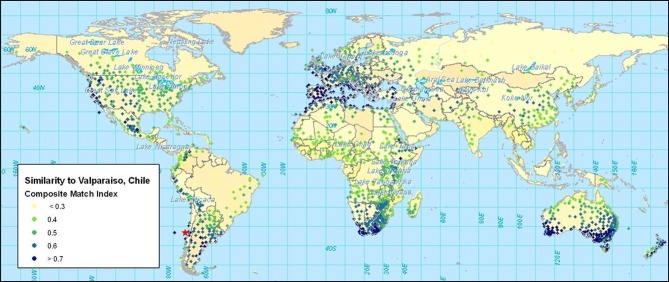
**The composite match index (CMI) showing the similarity of locations to Valparaiso, Chile**.

Once climate data have been standardized, comparisons can be made to evaluate the degree of similarity between any set of stations. The use of the climate-matching software CLIMEX illustrates this concept^5^. The software utilizes a large database of climate stations with 30 years of weekly data. CLIMEX displaces data to standardize them according to latitude. Difference equations are applied to create indices of similarity for maximum, minimum, and average temperature, rainfall, and rainfall pattern, humidity and soil moisture. Table 2 shows the results of a climate similarity analysis between Valparaiso, Chile, and four other stations in Mediterranean climates. Included here are stations with some of the highest similarity indices in the United States of America (USA), Australia, and the northern and southern extremes of Africa. Temperature and rainfall values are shown together with the similarity indices calculated by CLIMEX. The composite match index (CMI) combines the six climate parameters mentioned above. The corresponding map (Figure 2) shows the CMIs for over 2000 weather stations throughout the world. Higher CMI values indicate greater similarity.

**Table 2:** **The similarity of locations to Valparaiso, Chile: Temperature, rainfall, and similarity indices**^a^.

**Location**	*****T***_**min**_**	*****T***_**max**_**	*****R***_**tot**_**	***I*-***T***_**min**_**	***I*-***T***_**max**_**	***I*-***R***_**tot**_**	***CMI***
Valparaiso, Chile	8.3	22.2	506	1.00	1.00	1.00	1.00
Kingscote, Australia	8.2	24.8	485	0.87	0.86	0.97	0.88
San Francisco, USA	7.2	20.6	463	0.90	0.75	0.92	0.87
Wingfield, South Africa	7.2	26.1	509	0.82	0.68	0.99	0.86
Shahhat, Libya	4.4	28.3	608	0.69	0.59	0.87	0.79

a *T_min_, minimum temperature; T_max_, maximum temperature; R_tot_, total rainfall; I-T_min_, I-T_max_, and I-R_tot_ are the similarity indices. The CMI is a combination of similarity indices. For a description of the method, see Sutherst and Maywald (1991)*.

**Figure 3:**
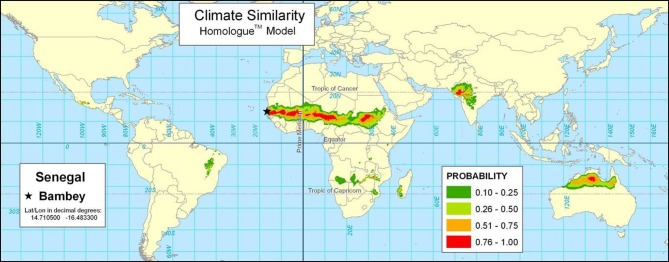
**The Homologue model showing areas similar in climate to Bambey, Senegal**.

Similarity analyses can be extended from weather station data to cover a continuous surface through spatial interpolation of climate data. For example, Figure 3 shows the result of the Homologue model for Bambey, Senegal. Homologue eliminates the need for input weather station data by interpolating climate data between stations^6^. The mapped results cover a continuous surface. The Bambey, Senegal station is similar to environments across the Sahel region of sub-Saharan Africa, and has been important in French efforts in agricultural research throughout West Africa. The map shows many areas that are right at the edge of very dry areas marginal for agriculture, such as northeast Brazil and the southern African area bordering the Kalahari desert.

The tools described above can be used for planning variety trials but lack information on the crop of interest. As discuss below, linking locations to the ecological niches of the crop of interest provides a more reliable basis for considering where a genotype could be targeted.

### Agro-ecological mapping

Maps of the systems characteristics, production, and ecology of crops can support the task of targeting genotypes to environments for both testing and deployment. Agricultural system maps draw on qualitative and quantitative information to depict regions of similar farming characteristics (Whittlesey, 1936; van Lanen et al., 1992; Pollack and Corbett, 1993; Dixon et al., 2001). We have already described the development of crop production maps. Maps of the ecology and environment of a particular crop are especially useful in targeting genotypes to environments.

In the latter part of the 1980s, an FAO workshop and resultant publication indicated a growing interest in environmental and agro-ecological mapping by the international agricultural research and development community (Bunting, 1987). Examples of this type of mapping work include CIAT's agro-ecological maps of cassava (CIAT, 2003) and rice (Jones, 1984). Such work often focused on regions instead of crops, and CIAT used these maps to define its research domain in Latin America (Jones et al., 1990). They were also used to assess the geographical distribution of environments that were the target of research in the Brazilian Cerrados (Jones et al., 1992).

**Figure 4:**
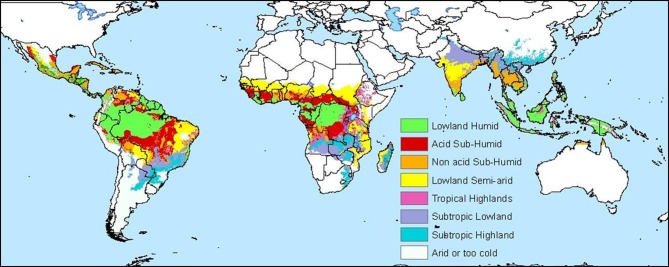
**Edapho-climatic map of cassava (CIAT, 2003)**.

**Figure 5:**
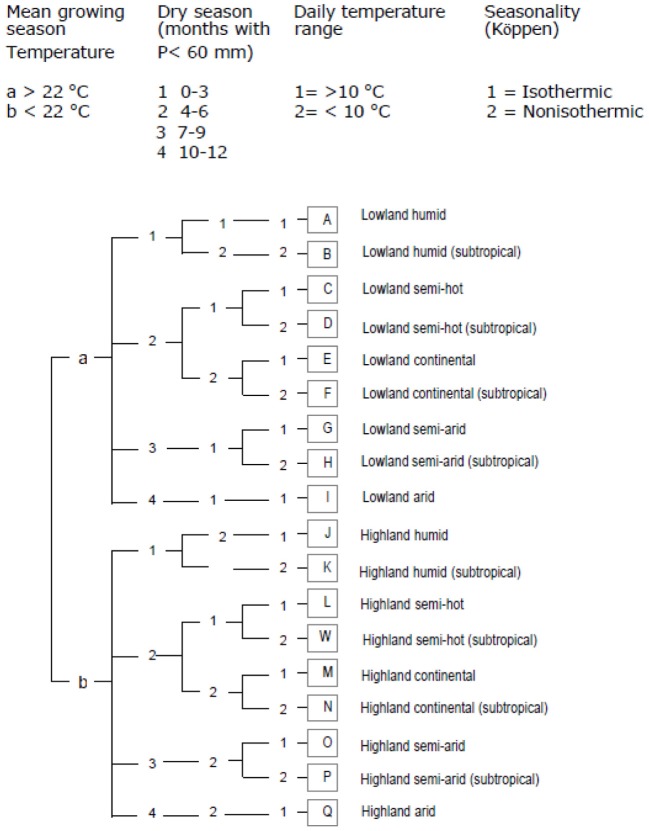
**Diagram showing the classification scheme for CIAT's cassava agro-ecology map (CIAT, 2003)**.

Methods for making these maps vary with respect to the type of data used and the statistical analyses employed. A cassava agro-ecology map is based on key precipitation, soil and elevation thresholds that define regions according to moisture conditions, soil acidity, and altitude (Figures 4 and 5; Carter, 1987; Carter et al., 1992; CIAT, 2003). For this classification system, cassava specialists identified key environmental thresholds for distinguishing between seven cassava agro-ecological regions. In a different approach, the Brazilian Cerrados was mapped using climatic and soils data in a cluster analysis (Jones et al., 1992). Clusters were mapped directly from the data and then generalized into homogenous regions within the Cerrados. A similar approach was carried out to map wheat agro-ecologies in Algeria using an unsupervised classification of GIS data layers (Delli et al., 2002).

The agro-ecological maps described above include knowledge of the crop but are not based on actual trial data. The discussion below turns to the CIMMYT methodology for mapping mega-environments, an approach that starts with the results of cultivar trials.

### Cimmyt's mega-environment approach for maize and wheat

**Figure 6:**
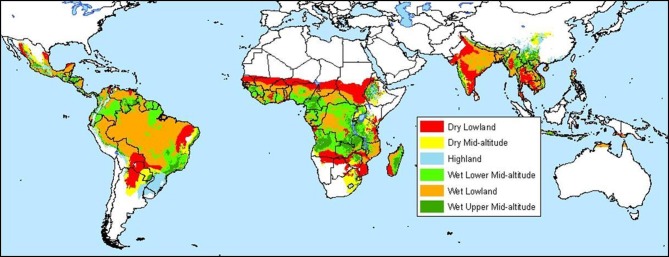
**Maize mega-environments**.

For maize, and specifically in the highly variable drought-prone environments of southern Africa, similar statistical techniques to multilocation yield trial data were applied, combined with environmental factors derived from GIS (Setimela et al., 2003, 2005; Maideni, 2006). Cluster analysis grouped the regional trials into seven groups with seasonal maximum temperature, precipitation, soil pH, and nitrogen stress identified as the factors accounting for repeatable GEI. Six final mega-environment zones were derived based on seasonal maximum temperature and precipitation, because available soil pH data were considered too unreliable for inclusion. Hence, maize germplasm in any mega-environment would have a requirement for evaluation under both low and high nitrogen and low and neutral pH. This combination of approaches has resulted in a better understanding of target environments in southern Africa (Bänziger et al., 2004) and has assisted in the identification of breeding strategies and key locations for regional variety testing. The stress factors responsible for GEI at the global scale were extrapolated and fine-tuned for southern Africa through feedback from experts (Figure 6).

For wheat, CIMMYT has developed mega-environments that have as a foundation the extensive network of international wheat testing sites, comprising over 800 unique sites. Wheat experts classified trial sites according to the predominant mega-environment and, subsequently, GIS was used to extract the underlying climatic and edaphic factors, resulting in quantitative criteria for mapping the mega-environments (Hodson and White, 2007). Long-term mean minimum temperature in the coolest quarter (i.e., three consecutive coolest months of the year) proved effective in distinguishing among the winter-grown spring, winter/facultative, and summer-grown spring wheat types. This temperature criterion was also useful for separating favorable, irrigated spring wheat environments from environments that are similar but where heat tolerance is required.

The climatic basis of both the maize and wheat mega-environments, and other agro-ecological mapping efforts, relies on long-term normal data such as Worldclim, described in the introduction (Hijmans et al., 2005). While the approaches have improved the understanding of general crop agro-ecologies, they ignore temporal variation due to year-to-year variation in climatic conditions. Trethowan et al. (2005) showed how specific locations may fluctuate between high or low rainfall wheat mega-environments depending on seasonal conditions. Such limitations are now being addressed by work on frequencies of environment types.

In practical terms, the real nature of the problem from the point of view of GEI is that testing environments may represent the wrong balance of stress intensity or timing, so selection will not address optimally the needs of the target population of environments (TPE). In highly variable environments, the degree of mismatch between the sample from multienvironment trials and the TPE is likely to be high, and could lead to decreased or even reversed genetic gain (Cooper et al., 1996).

Considerable advances are being made in the area of improved characterization of TPE, environment types, and frequencies of environment types. These advances are largely due to the coupling of crop simulation models with long-term weather records in order to generate seasonal sequences of stress that can subsequently be used to determine frequencies of stress environment types (Chapman and Barreto, 1996; Hartkamp et al., 1999, 2001; Chapman et al., 2000a, 2002, 2003; Loffler et al., 2005; Putto et al., 2009). This type of information, in combination with multienvironment trial data, can be used to weight data from different trials according to how representative they are of the TPE and so improve selection, especially in variable environments (Chapman et al., 2000b).

Loffler et al. (2005) used the crop simulation and GIS approach to classify the major maize environments in the Corn Belt of the USA. Even in this highly productive maize environment, the spatial and temporal dimensions of environmental variation in the TPE were highly significant. For each of the six major environment types identified, relative frequencies of each of the environments varied greatly from year to year and significant hybrid by environment interaction variance was observed. Stratification of environments sampled by the multienvironment trials by the temporally specific environment type explained a significant portion of the GEI for observed grain yield. This methodology is therefore likely to improve the predictability of cultivar performance in the TPE. These new approaches have only been reported from the USA or Australia but future application to highly variable environments such as Africa have the potential to produce significant breeding gains.

### Spatial analysis of environmental change

Projections of environmental change are motivating greater emphasis on future constraints to agricultural production. The pace of population, climatic, and environmental change has compelled the crop improvement community to consider those stresses that are likely to result in significant yield declines (Cassel-Gintz et al., 1997). Spatial analysis is already playing a role in assisting breeding programmes to respond to environmental change. The rapid changes in soils and climate will likely increase this role in the coming decades.

Intensive land use and agricultural development erode, leach, and degrade our soils. In the absence of improved agronomic practices and land management, cultivars of the future will probably need to be tolerant of aluminium toxicity, low nutrient status and other chemical changes that make soils less fertile. Salinisation of soils will demand salt-tolerant cultivars. New varieties will have to survive in poorly structured soils with low water-holding capacity. Crop improvement to overcome abiotic soil constraints will focus on these difficult soil environments. Crop improvement specialists can map accessions of wild relatives overlaid on environmental stresses to provide clues about which accessions may be adapted to a given stress. More cultivar testing needs to be carried out in those soil environments where a particular production constraint is representative of the growing soil problems we shall face in the future. However, improved agronomic practices will play a vital mitigating role and these need to be an integrated part of crop improvement.

Of more immediate concern for crop improvement are the effects of climate change (Jones and Thornton, 2003; Lobell et al., 2008). Improved cultivars have a product life cycle (research, development, testing and use) of 46 years on average (Jones et al., 2007b). Therefore, the development of new cultivars should aim for adaptation in the climate we will find in 30–50 years from now. For example, an analysis of testing sites for biofortification programmes found that many of the current maize testing sites in Africa do not represent the likely environments for maize in 2055 (Jones et al., 2007b). Another important consideration for crop improvement is the conservation of wild relatives and landraces that may otherwise become extinct due to climate change. Jarvis et al. (2003) found that of 17 wild *Arachis* species in South America, 12 could be extinct in 50 years time due to climate change. If we do not conserve these genetic resources now, future efforts may lack valuable material needed for crop improvement.

New data and tools are facilitating spatial analysis of climate change. Downscaled weather data from General Circulation Models are often used in modeling climate change impacts on agriculture (Jones and Thornton, 2013). The Worldclim data set now includes downscaled projections of future climate for three popular climate models from the Intergovernmental Panel on Climate Change (IPCC) family of climate change scenarios^7^. These data can be used directly in GIS software packages such as DIVA (Hijmans et al., 2001). Initial efforts have been made to incorporate future climate projections in CIAT's Homologue and Marksim tools. Researchers of the CGIAR have downscaled the 21 IPCC model scenarios for climate change to 1-km climate surfaces, some of which have not been publicly released to date. These recent developments suggest that the prospects for using spatial analysis for studies of genetic resources and climate change are improving.

## Conclusion

Methods to target genotypes to environments are evolving. Plant breeders used a “hit-or-miss” approach for many years, simply testing their cultivars in as many environments as they could. The development of agro-ecological mapping (as per Bunting, 1987) gave them a better idea about the target environments. Developing maps from large international yield trials, as in CIMMYT's mega-environment approach, improved on agro-ecological mapping. Spatially explicit crop modeling has improved targeting studies over the last decades. Recent efforts to account for changes in year-to-year environmental conditions have further improved our understanding of how to more efficiently reach our goal of getting the right genotype to farmers.

Geographic information science and technology has played a valuable role in the evolution of genotype targeting approaches. It has provided high-resolution spatial and temporal data to help breeders unravel GEI. Spatial synthesis of model and statistical outputs has improved our capacity to map out target environments and the frequencies of environments, an effort that ultimately leads to a more effective deployment of germplasm. Greater collaboration between breeders, crop improvement specialists, and the climate change modeling community are needed now more than ever.

### Conflict of interest statement

The authors declare that the research was conducted in the absence of any commercial or financial relationships that could be construed as a potential conflict of interest.

## Footnotes

^1^http://earlywarning.usgs.gov/adds/index.php?img1=rf&extent=af

^2^http://ingrid.ldeo.columbia.edu/maproom/.Food_Security/.Locusts/index.html

^3^http://www.figstraitmine.org/index.php?dpage=11

^4^ftp://ftp.ncdc.noaa.gov/pub/data/gsod

^5^http://www.hearne.com.au/products/climex/

^6^Contact article author Glenn Hyman (g.hyman@cgiar.org) to request Homologue software.

^7^http://www.worldclim.org/futdown.htm
